# The Danger of Keeping Dogs

**Published:** 1888-01

**Authors:** 


THE DANGER OF KEEPING DOGS.—One of the dangers resulting from keeping dogs in dwelling houses is the risk of disseminating the ova of the *tænia echinococcus.* The possibility of the transmission of this dangerous insect is too generally overlooked, but if the dog play with the children, the latter often allow themselves to be licked, and in this way the ova may be transferred. In dry weather the ova may be wafted about by the wind, and so find their way into the body. In Iceland, where everybody possesses half-a-dozen dogs, 28 per cent. of which are affected with this tænia, hydatid cysts are very common. As the process of development is a slow one, the source of the infection may, and probably will, escape attention, and in any case would only be thought of when the evil had been done.—*Medical Press and Circular.*

